# Procalcitonin or lactate clearance, or both, for risk assessment in patients with sepsis? Results from a prospective US ICU patient cohort

**DOI:** 10.1186/cc14139

**Published:** 2015-03-16

**Authors:** N Braun, P Schuetz, R Baruti, D Amin

**Affiliations:** 1 Kantonsspital Aarau, Switzerland; 2 Morton Plant Hospital, Clearwater, FL, USA

## Introduction

Blood lactate level is a routinely used biomarker for management of patients in septic conditions in the ICU. There is a lack of clinical research data comparing lactate with novel sepsis biomarkers, such as procalcitonin, in regard to the diagnostic and prognostic potential. Herein, we investigated the diagnostic and prognostic value of initial lactate and procalcitonin levels and their kinetics within the ICU stay for prediction of positive blood cultures and fatal outcome in a well characterized cohort of sepsis patients in a US critical care setting.

## Methods

This is a retrospective, observational cohort study of adult patients with confirmed severe sepsis or septic shock and with at least one procalcitonin and lactate measurement on admission to the ICU of Morton Plant Hospital (Clearwater, FL, USA). Logistic regression models were calculated to assess the association of biomarkers with blood culture positivity and fatal ICU outcome with area under the curve (AUC) as a measure of discrimination.

## Results

The in-hospital mortality rate of the 1,075 included patients (age 68 years) was 23.8% (95% CI = 21.2 to 26.3%) and 18.4% of patients had positive cultures. In regard to the diagnostic value for bacteremic disease, initial procalcitonin had a higher discriminatory value (AUC 0.71) compared with initial lactate levels (AUC 0.52). In regard to prognosis, initial lactate level was the better mortality predictor (AUC 0.69) compared with procalcitonin (AUC 0.55), although both initial levels were significantly lower compared with APACHE III (*P *> 0.05 for both comparisons). When looking at biomarker kinetics, procalcitonin decrease was more strongly associated with fatal outcome compared with initial levels alone (AUC 0.66), but still lower compared with lactate kinetics (AUC 0.73).

## Conclusion

Both biomarkers, procalcitonin and lactate provide diagnostic and prognostic information in ICU patients with sepsis, particularly when looking at biomarker kinetics. An evidence-based protocol incorporating both markers may further improve management of septic patients.

